# Neurological assessment of newborns with spinal muscular atrophy identified through neonatal screening

**DOI:** 10.1007/s00431-022-04470-3

**Published:** 2022-05-06

**Authors:** Marika Pane, Maria Alice Donati, Costanza Cutrona, Roberto De Sanctis, Matteo Pirinu, Giorgia Coratti, Martina Ricci, Concetta Palermo, Beatrice Berti, Daniela Leone, Chiara Ticci, Michele Sacchini, Margherita Cerboneschi, Anna Capasso, Gianpaolo Cicala, Maria Carmela Pera, Chiara Bravetti, Emanuela Abiusi, Alessandro Vaisfeld, Giovanni Vento, Francesco Danilo Tiziano, Eugenio Mercuri

**Affiliations:** 1 Centro Clinico Nemo Pediatrico, Fondazione Policlinico “A. Gemelli” IRCCS, Rome, Italy; 2 Pediatric Neurology Unit, Università Cattolica del Sacro Cuore, Largo Gemelli, Rome, Italy; 3 Metabolic and Muscular Unit, Meyer Children’s Hospital, Florence, Italy; 4 Rehabilitation Unit, Meyer Children’s Hospital, Florence, Italy; 5 Department of Life Sciences and Public Health, Section of Genomic Medicine, Fondazione Policlinico “A. Gemelli” IRCCS, Università Cattolica del Sacro Cuore, Rome, Italy; 6 Unit of Neonatology, Fondazione Policlinico Universitario “A. Gemelli” IRCCS, Università Cattolica del Sacro Cuore, Rome, Italy

**Keywords:** Neonatal, Screening, SMA, Assessment

## Abstract

The possibility to identify patients with spinal muscular atrophy through neonatal screenings has highlighted the need for clinical assessments that may systematically evaluate the possible presence of early neurological signs. The aim of this study was to use the Hammersmith Neonatal Neurological Examination (HNNE) and a module specifically designed for floppy infants to assess the possible variability of neurological findings in infants identified through neonatal screening. The infants included in this study were identified as part of a pilot study exploring neonatal screening in two Italian regions. A neurological examination was performed using the HNNE and an additional module developed for the assessment of floppy infants. Seventeen infants were identified through the screening. One patient had 1 SMN2 copy, 9 had 2 copies, 3 had 3, and 4 had more than 3 copies. Nine of the 17 infants (53%) had completely normal results on both scales, 3 had minimal signs, and the other 5 had more obvious clinical signs. The number of *SMN2* copies was related to the presence of abnormal neurological signs (*p* = 0.036) but two *SMN2* copies were associated with variable clinical signs as they were found in some infants with respectively normal examination or obvious severe early signs.

*Conclusion*s: Our results suggest that the combination of both scales increases the possibility to detect neonatal neurological signs and to define different early patterns of involvement also identifying paucisymptomatic patients.

**Table:** 

**What is Known:** *• The use of new therapeutic options in presymptomatic SMA patients leads to a dramatic reduction of the onset and severity of the diesease.* *• The already existing tools commonly used in Type I SMA (HINE and CHOP-intend) may not be suitable to identify minor neurological signs in the neonatal period.*	
**What is New:** *• Combining the HNNE and the floppy infant module, we were able to identify early neurological signs in SMA infants identified through newborn screening and may help to predict the individual therapeutic outcome of these patients.* *• Iinfants with 2 SMN2 copies identified through the screening had a more variable neonatal examination compared to those with three or more copies, in agreement with similar findings in older infants.*

**Supplementary information:**

The online version contains supplementary material available at 10.1007/s00431-022-04470-3.

## Introduction

The advent of disease modifying therapies has dramatically changed the field of spinal muscular atrophy (SMA) [1]. In the last 5 years, three different approaches targeting the increase of the survival motor neuron (SMN) protein have become available following successful clinical trials. One of these approaches is based on gene replacement (Onasemnogene Abeparvovec) with clinical trials targeting young type 1 infants [2]. The other two approaches, based on *SMN2* splicing through antisense oligonucleotides (Nusinersen) [3, 4] or a small molecule (Risdiplam) [5, 6], have become available for all types and ages of SMA. Recent studies have clearly demonstrated that the use of these new therapeutic options in infants before the onset of symptoms leads to a dramatic reduction of the onset and severity of clinical signs with a magnitude of improvement much larger than that observed in patients who had already developed symptoms at the time of treatment [7]. In a phase II, open-label, single-arm study using Nusinersen in 25 pre-symptomatic SMA infants approximately 90% of the treated presymptomatic patients became able to sit and walk independently at the same age when typically developing children acquire these milestones (up to 9 and 18 months, respectively) [7].

The use of Onasemnogene abeparvovec and Risdiplam in presymptomatic patients have also been reported in recent congresses with most infants also achieving age appropriate milestones. These results have further highlighted the need for neonatal screening (NBS) that is increasingly becoming available in several Countries [8–11]. A few studies have recently reported regional or national screening programs reporting variable percentages of infants who already had obvious clinical signs in the first weeks, consistent with a diagnosis of type 1 [8–11]. In other infants, only minor signs such as weak or absent reflexes or mild truncal hypotonia occurred even in the absence of major obvious signs [8, 12]. There is therefore increasing evidence that a number of infants identified from the screening programs may be “paucisymptomatic,” as also suggested by some minor signs detected in clinical trials [2, 7]. These minor signs would have possibly not been detected on a quick routine neonatal check at discharge if a diagnosis had not been achieved with the screening. These findings have raised the issue of the suitability of the existing tool to identify minor neurological signs in the neonatal period as the scales commonly used in type 1 SMA such as the Hammersmith Infant Neurological Examination (HINE) [13] or the Children’s Hospital of Philadelphia Infant Test of Neuromuscular Disorders (CHOP INTEND) [14] are validated for older infants.

The aim of this study was to use structured neurological examinations assessing different aspects of neurological function, including a module specifically designed for floppy infants, in order to assess the possible variability of neurological findings in a cohort of patients identified through neonatal screening.

## Subjects and methods

The infants included in this study were identified as part of a pilot study exploring neonatal screening in two large Italian regions (Lazio and Toscana). As part of this study, all parents of infants born in the participating hospitals were offered with the possibility to have screening for SMA performed in their children. Following the identification of *SMN1* homozygous absence in the high-throughput process, patients were re-sampled for confirmation of the diagnosis. The following analyses were performed on fresh blood samples: confirmation of *SMN1* deletion, *SMN2* copy number assessment by real-time PCR, search of splicing variants c.859G > C and c.-44A > G (rs121909192 and rs1454173648, respectively) by Sanger sequencing.

Patients were all assessed with a structured neurological assessment on their first visits and if already symptomatic, were classified as type 1 as they had onset within 6 months with severe hypotonia, weakness, and the typical respiratory pattern. Infants who also had marked reduced fetal movements, contractures, and very severe respiratory impairment at birth suggestive of antenatal onset were classified separately as Type 0.

The neurological examination was performed using a structured neurological examination (HNNE) [15] and an additional module developed for the assessment of floppy infants [16] focused on the identification of neuromuscular signs.

### HNNE

The HNNE is performed and recorded using a standardized form including 34 items grouped in six categories (posture and tone, tone patterns, reflexes, movements, abnormal signs/patterns, and orientation and behavior).

Each item consists of numbered columns, between 3 and 5, with diagrams and drawings providing different options. The option that corresponds most closely to the infant’s response to the test item is circled, identifying the column 1, 2, 3, 4, or 5 in which it fell. If an item falls between 2 columns, it is given the appropriate half score between the two columns (e.g., Items scoring between 1 and 2 scored 1.5). These scores are defined as raw scores and can be converted into an optimality score based on the frequency distribution of the findings for each item in a low risk cohort, providing the opportunity to define optimality for individual items and for the global examination [15]. The 10th centile was used as a cut-off point to define as non-optimal findings that were not frequent in the low risk cohort.

### Floppy infant module

The additional module for floppy infants includes a section on neurological aspects, one on physical examination and one on additional information such as antenatal history that can help to identify signs suggestive of neuromuscular disorders. The use of this module allows to recognize the signs that are specific of 1 SMA, i.e., the typical posture with intrarotated arms, the pattern of weakness and hypotonia (lower limb > upper limb, proximal > distal), the diaphragmatic breathing pattern, together with absent reflexes and fasciculations. Findings can be documented on the form in one of three columns: the third column includes the findings that are considered optimal, the first column findings that are known to be often abnormal, and the second column intermediate findings that require surveillance. The examinations were performed by one or two examiners in each center, who had all been trained by the same person with formal assessments of inter-observer reliability. The examinations were also videoed and rescored by two examiners.

### Statistical analysis

Demographic and clinical characteristics were summarized using frequencies (percentage) for categorical variables.

The cohort was subdivided into *SMN2* copy number (≤ 2 and > 2) and diagnostic criteria after neurological examination. We labeled as *asymptomatic* the infants with no abnormal neurological sign; *paucisymptomatic* if they had isolated abnormal signs, and symptomatic patients if they had a more wider neurological impairment. *Symptomatic* patients were further subdivided into *specific*, if they had the typical pattern of neurological signs consistent with type SMA( see methods), or *non-specific*, if they had more than isolated signs but not the typical SMA pattern. We also separated type 0 patients who have a distinct pattern of impairment with contractures and other signs related to antenatal involvement.

To run statistical test, due to sample size, we re-subgrouped patients in “asymptomatic” and “symptomatic” including all patients with abnormal findings from mild to severe, under this category. Chi-square test was used to analyze distribution of patients by *SMN2* copies and HNNE findings (normal, abnormal). All data processing steps and statistical analysis was performed in SPSS version 27.

## Results

Seventeen patients were identified through the screening. With the exception of the infant with prenatal abnormal signs, all infants were born at term age, all with normal Apgar scores, with birthweights appropriate for gestational age. Thirteen patients (76.4%) were born by spontaneous vaginal delivery and four (23.5%) by elective cesarean delivery. No perinatal complications were reported from all patients, with the exception of SMA 0 patient, who showed absence of spontaneous breathing at birth and required immediate tracheal intubation and mechanical ventilation.

All patients received final confirmation of the diagnosis before the age of 2 weeks and were assessed at the time of first consultation (date of examination range 3–13 days after birth). One patient had 1 SMN2 copy, 9 had 2 copies (1 of the 9 also with the heterozygous c.859G > C), 3 had 3 copies, and 4 had more than 3 copies. Five patients, all with 2 SMN2 copies, had overt clinical neurological signs at birth, 4/5 were classified as SMA 1 while 1/5 was classified as SMA 0 since he had prenatal symptom onset and respiratory failure at birth.

### HNNE

**Fig. 1:**
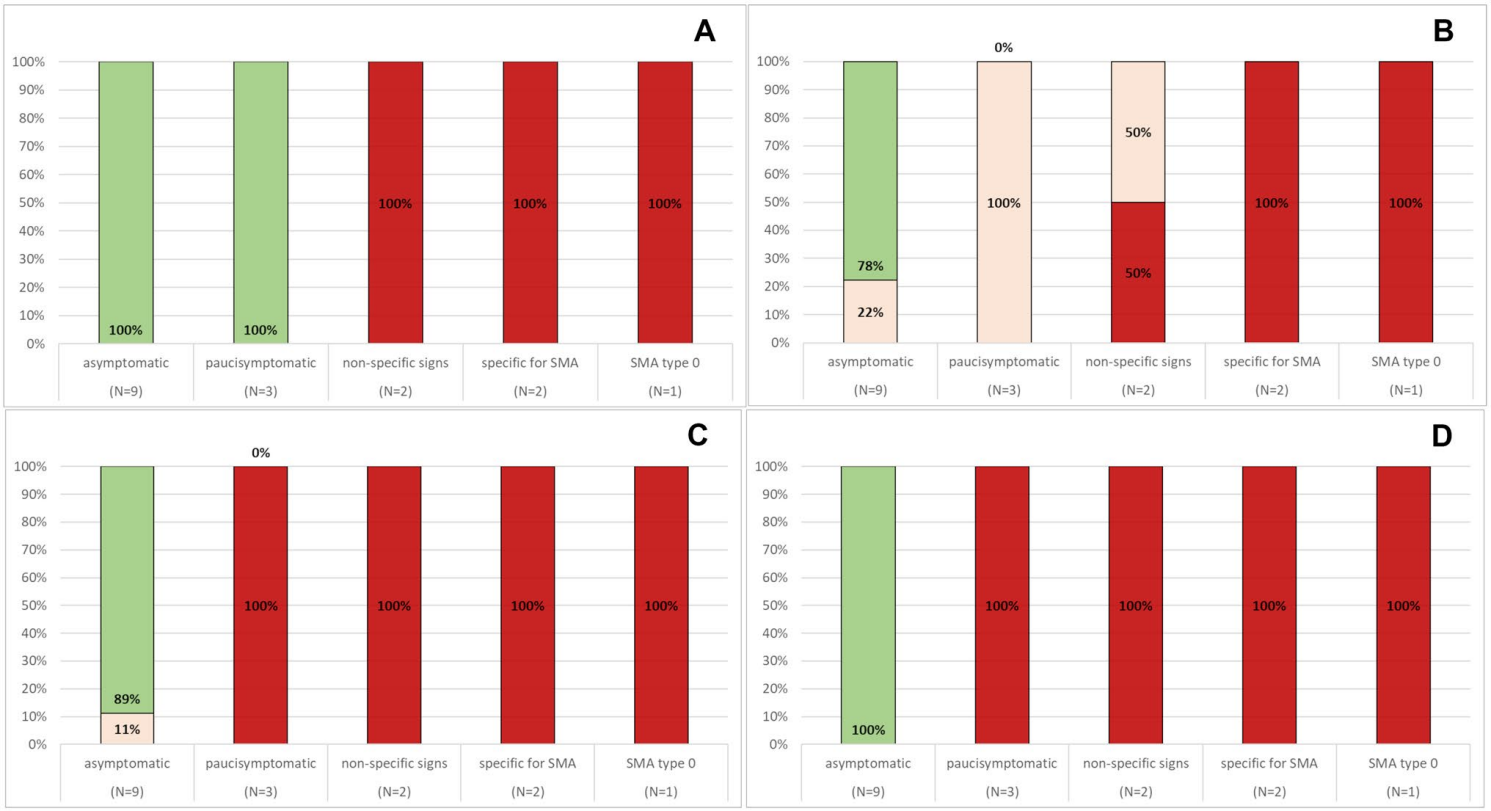
HNNE tone and posture optimality score. The figure shows details of the frequency of optimality scores in the individual item in the Tone and Posture section. Panel **A** shows an example of the recurrent pattern of optimality observed in many items, with asymptomatic and presymptomatic infants all having optimal results and symptomatic patients all having suboptimal results. This pattern was found in the items: arm recoil, arm traction, leg traction, popliteal angle, head control (1), head control (2) and head lag. The other panels show items who had different patterns of responses: posture (**B**); leg recoil (**C**); ventral suspension (**D**)

**Fig. 2:**
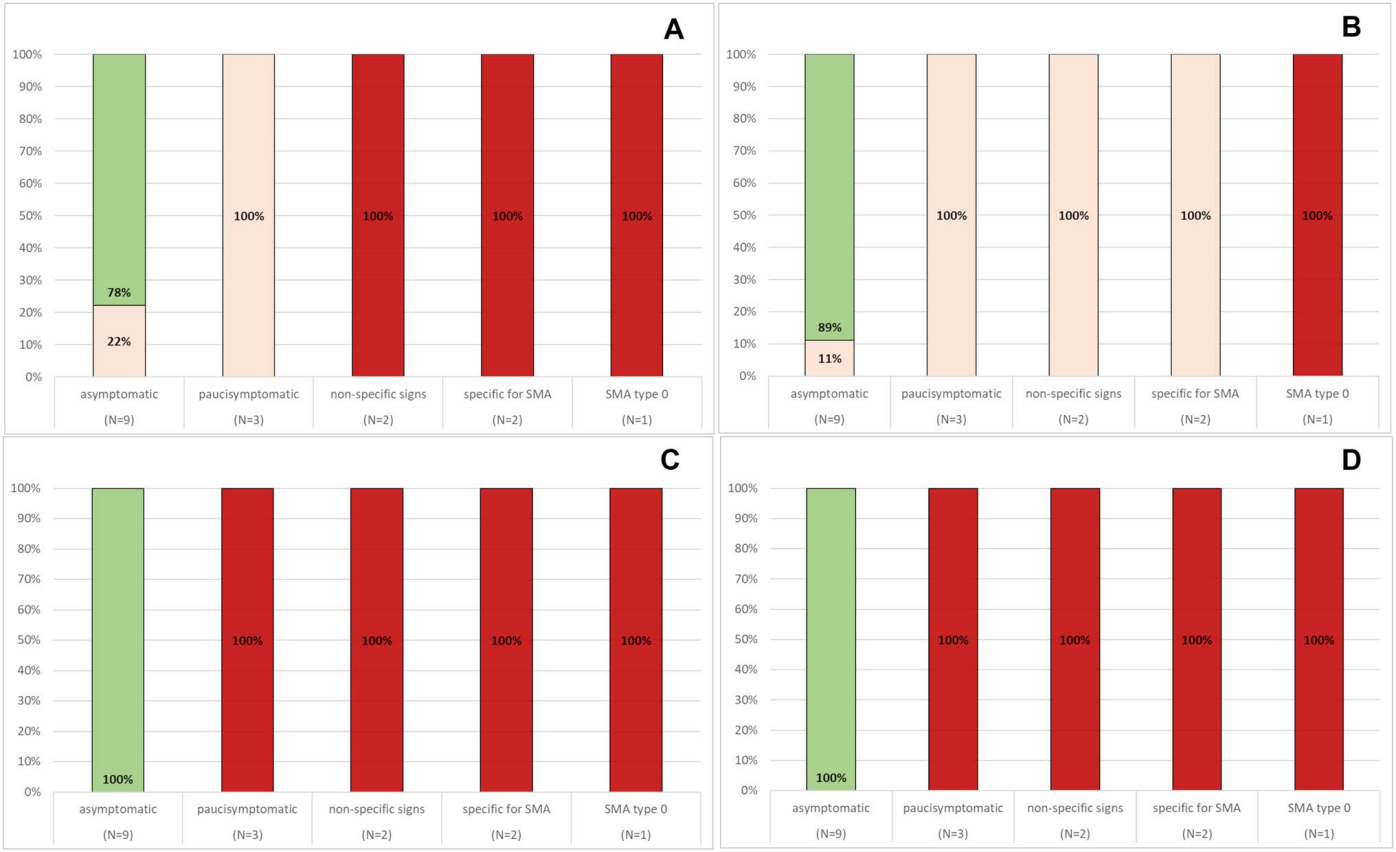
HNNE movements and reflexes optimality scores. The figure shows details of the frequency of optimality scores in the individual items assessing: quantity (**A**) and quality (**B**) of Spontaneous movements; Head raising (prone) (**C**). Panel **D** shows the frequency of optimality scores for tendon reflexes

The global HNNE scores were optimal in 12 patients and suboptimal in 5. Figures 1 and 2 show details of the optimality scores for individual items. The items that were more frequently suboptimal in the whole cohort were posture, leg recoil, ventral suspension, and reflexes. Behavioral items were normal in all patients, irrespective of their motor signs.

### HNNE and SMN2 copies

The distribution of *SMN2* copy number between normal and abnormal findings on the HNNE was significantly different (*X*^2^ (1, *N* = 16) = 4.390, *p* = 0.036), with patients with ≥ 3 copies having more optimal results on the HNNE than those with ≤ 2 SMN2 (66.7% vs 14.3%), and, as a consequence, less optimal results (33.3% vs 85.7%).

### Floppy infant module

Nine patients had normal results on all items, three had abnormal results on two items, and the remaining five had more than 2 abnormal results. Table 1 show details of the distribution of individual items on the floppy module in the subgroups.

**Table 1:** Distribution of individual items on the floppy module in the subgroups

		Asymptomatic Patients (*N* = 9)		Paucisymptomatic Patients (*N* = 3)		Patients with non-specific SMA signs(*N* = 2)		Patients with specific SMA signs(*N* = 2)		Patient with presentation consistent with SMA 0(*N* = 1)		All patients(*N* = 17)	
		*N*	%	*N*	%	*N*	%	*N*	%	*N*	%	*N*	%
Antigravity movements Lower limbs	*Normal*	9	100%	3	100%	0	0%	0	0%	0	0%	12	71%
	*Intermediate*	0	0%	0	0%	2	100%	2	100%	0	0%	4	24%
	*Abnormal*	0	0%	0	0%	0	0%	0	0%	1	100%	1	6%
Antigravity movements Upper limbs	*Normal*	9	100%	3	100%	0	0%	0	0%	0	0%	12	71%
	*Intermediate*	0	0%	0	0%	2	100%	2	100%	0	0%	4	24%
	*Abnormal*	0	0%	0	0%	0	0%	0	0%	1	100%	1	6%
Tendon reflexes	*Normal*	9	100%	0	0%	0	0%	0	0%	0	0%	9	53%
	*Intermediate*	0	0%	2	67%	1	50%	0	0%	0	0%	3	18%
	*Abnormal*	0	0%	1	33%	1	50%	2	100%	1	100%	5	29%
Contractures Lower limbs	*Normal*	9	100%	3	100%	1	50%	0	0%	0	0%	13	76%
	*Intermediate*	0	0%	0	0%	1	50%	1	50%	0	0%	2	12%
	*Abnormal*	0	0%	0	0%	0	0%	0	0%	1	100%	1	6%
Contractures Upper limbs	*Normal*	9	100%	3	100%	2	100%	2	100%	0	0%	16	94%
	*Intermediate*	0	0%	0	0%	0	0%	0	0%	0	0%	0	0%
	*Abnormal*	0	0%	0	0%	0	0%	0	0%	1	100%	1	6%
Facial muscles	*Normal*	9	100%	3	100%	2	100%	2	100%	0	0%	16	94%
	*Intermediate*	0	0%	0	0%	0	0%	0	0%	1	100%	1	6%
	*Abnormal*	0	0%	0	0%	0	0%	0	0%	0	0%	0	0%
Respiratory pattern	*Normal*	9	100%	3	100%	0	0%	0	0%	0	0%	12	71%
	*Intermediate*	0	0%	0	0%	2	100%	2	100%	0	0%	4	24%
	*Abnormal*	0	0%	0	0%	0	0%	0	0%	1	100%	1	6%
Sucking	*Normal*	9	100%	3	100%	1	50%	2	100%	0	0%	15	88%
	*Intermediate*	0	0%	0	0%	1	50%	0	0%	0	0%	1	6%
	*Abnormal*	0	0%	0	0%	0	0%	0	0%	1	100%	1	6%
Swallowing	*Normal*	9	100%	3	100%	1	50%	2	100%	0	0%	15	88%
	*Intermediate*	0	0%	0	0%	1	50%	0	0%	0	0%	1	6%
	*Abnormal*	0	0%	0	0%	0	0%	0	0%	1	100%	1	6%
Reduced fetal movements	*Normal*	9	100%	3	100%	2	100%	2	100%	0	0%	16	94%
	*Intermediate*	0	0%	0	0%	0	0%	0	0%	0	0%	0	0%
	*Abnormal*	0	0%	0	0%	0	0%	0	0%	1	100%	1	6%
Amniotic fluid	*Normal*	9	100%	3	100%	2	100%	2	100%	0	0%	16	94%
			0%		0%	0	0%	0	0%		0%	0	0%
	*Abnormal*	0	0%	0	0%	0	0%	0	0%	1	100%	1	6%
Seizures	*Normal*	9	100%	2	67%	2	100%	2	100%	1	100%	16	94%
	*Abnormal*	0	0%	0	0%	0	0%	0	0%	0	0%	0	0%
Dysmorfisms	*Normal*	9	100%	2	67%	2	100%	2	100%	1	100%	16	94%
	*Abnormal*	0	0%	0	0%	0	0%	0	0%	0	0%	0	0%
Other organ involvement	*Normal*	9	100%	2	67%	2	100%	2	100%	1	100%	16	94%
	*Abnormal*	0	0%	0	0%	0	0%	0	0%	0	0%	0	0%
Additional signs				*Fasciculations (n=2)*				*Fasciculations (n=1)*		*Fasciculations (n=1)*		Fasciculations *(n=4)*	

### Classification according to neurological findings

Nine patients had normal overall examination on both HNNE and floppy module and were classified as asymptomatic.

Three patients had isolated abnormal findings on the HNNE and/or on the floppy module, and were labeled as paucisymptomatic. Reflexes and fasciculations were more often abnormal with all infants also showing one or more suboptimal scores on posture, leg recoil and/or ventral suspension (Fig. 1).

Five had obvious abnormal neurological signs on HHNE suboptimal scores in most tone and reflex items. The floppy module items, assessing weakness and contractures, were abnormal in all patients. The distribution of weakness and the respiratory pattern allowed to identify specific signs of SMA in 3 of the 5 infants, with one of the three also showing antenatal onset and clinical signs consistent with type 0 SMA. The remaining two had non-specific signs of neuromuscular involvement.

### Neurological findings and SMN2 copy number

**Fig. 3:**
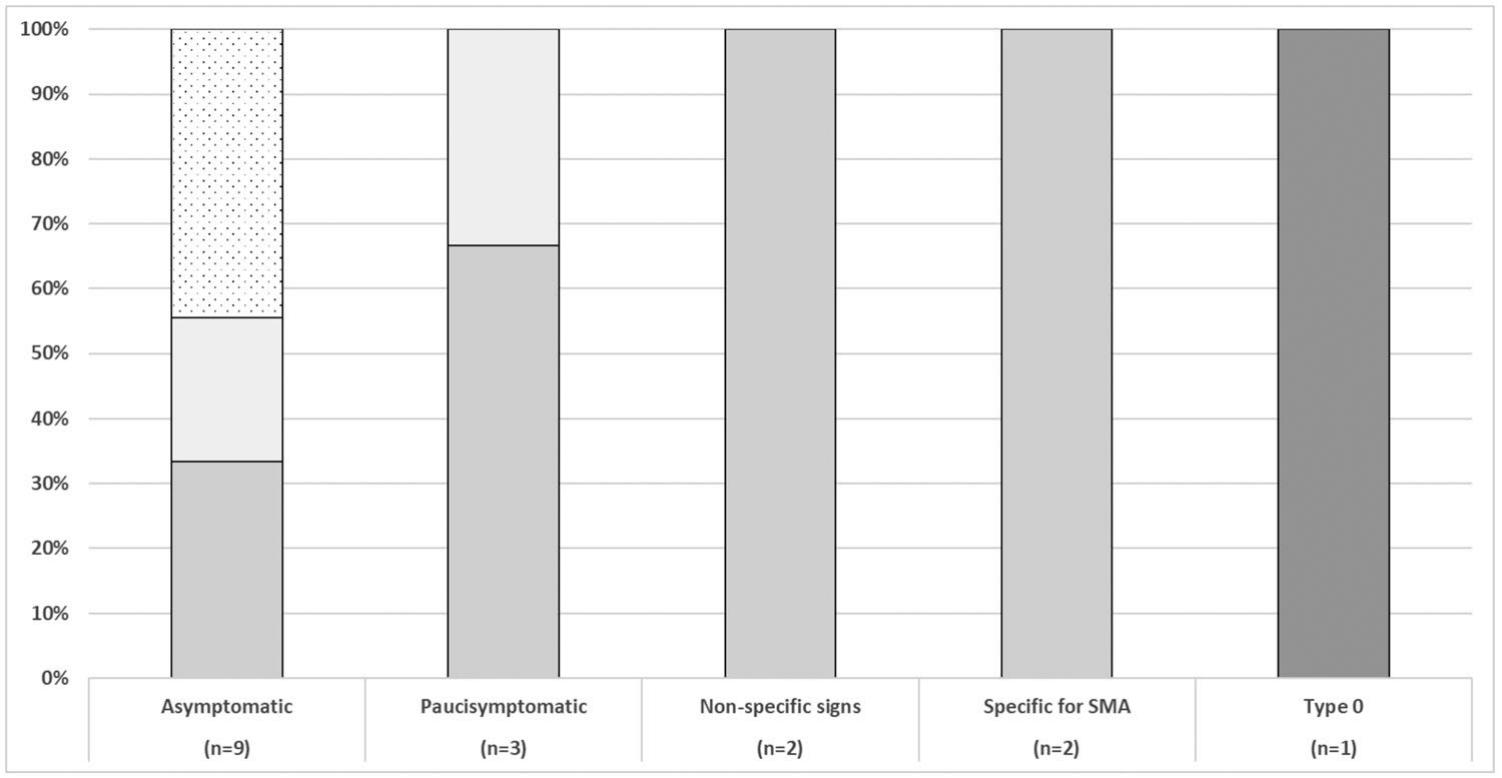
Correlation between SMN2 copies and neurological findings. Key to figure: *Dark gray*: patients with 1 SMN2 copy; *Gray:* patients with 2 SMN2 copies; *Light Gray*: patients with 3 SMN2 copies; *Dotted*: patients with more than 3 copies

Figure 3 shows details of the correlation between SMN2 copies and neurological findings.

## Discussion

The aim of this study was to explore the suitability of the HNNE and of the supplementary module specifically designed for floppy infants, to detect possible neurological signs in SMA infants identified through NBS. The HNNE is widely used in neonatology and is an easy, quick tool to collect several aspects of neurological function using a structured preform [15]. The recent floppy infant module has been developed as an add on module to capture more specific aspects of possible peripheral involvement [16]. Using our combined assessments, we found that only 9 of the 17 infants (53%) in our cohort had completely normal assessments. The remaining 47% had some abnormal findings in the first days, ranging from minimal clinical signs to the obvious severely abnormal signs consistent with a diagnosis of type 0 SMA [17].

Each of the two assessments helped to better document the clinical signs in these patients. While the HNNE, designed as a general neurological assessment for newborns, was able to detect all cases with obvious or minimal clinical signs, the floppy module provided a more qualitative assessment providing the opportunity to subdivide patients according to the presence or absence of SMA specific signs, that cannot be extrapolated by the HNNE. More specifically, the floppy module provides the possibility to identify the breathing paradoxical pattern and the presence of the typical SMA distribution of weakness with predominant involvement of lower limbs and proximal muscles. Other items, including those related to prenatal findings allowed to better identify different phenotypes in our cohort.

One infant had a type 0 SMA with prenatal onset and severe neonatal signs including contractures. Two further infants had early neonatal signs suggestive of SMA but no contractures or others signs indicative of antenatal onset, therefore consistent with a diagnosis of type 1 [18]. In the other infants, the neurological findings were not fully SMA specific as they had hypotonia and mild weakness with partial antigravity movements and weak reflexes, suggestive of a possible neuromuscular involvement. However, because of the absence of the typical weakness distribution, paradoxical breathing pattern or fasciculations, the diagnosis of SMA was not obvious in the absence of the NBS, thus differential diagnoses with other neuromuscular disorders had been taken into account.

Our results also confirmed that even in the absence of obvious clinical signs, a number of infants classically defined as presymptomatic, may have isolated minimal signs, such as hypotonia, tongue fasciculations, and weak/absent reflexes. The significance of weak reflexes may be underestimated during the routine discharge assessment as this sign can be found in over 20% of low-risk term infants [15]. In SMA patients identified through screening, however, reflexes should be carefully followed, as recent longitudinal case reports in untreated presymptomatic patients suggest that a reduction in tendon reflexes is the earliest clinical sign to appear, preceding of a few days/weeks the appearance of the full SMA clinical pattern [19]. The other suboptimal items at HNNE evaluation in paucisymptomatic patients were posture and lower limb tone (leg recoil) that had already been reported in other assessments in presymptomatic patients [8]. Interesting to note that in all three paucisymptomatic patients, we also found incomplete ventral suspension on the HNNE with an obvious discrepancy between extensor and flexor tone, as the head lag item was optimal. In one case, the child was able to achieve the full response but not to hold it for more than few seconds.

The number of *SMN2* copies was related to the presence of abnormal neurological signs. At the two extremes, 1 *SMN2* copy was found in the infants with type 0 while, with one exception, infants with three or more copies had normal results on both tools. Two *SMN2* copies in contrast were associated with variable clinical signs, as they were found in all the patients with overt minimal or severe clinical signs but also in one-third (3 out of 9) of the patients with normal examination. These findings suggest that patients with 2 copies are at higher risk of showing early abnormal signs but also confirm previous suggestions that the copy number cannot always predict the severity of the phenotype in individual cases [20].

One of the possible confounding factors to explain variability is related to the presence of the c.859C > G rare *SMN2* polymorphisms affecting exon 7 alternative splicing, since this variant has been retrospectively found only in subjects with milder forms of SMA [20]. In agreement with previous reports, the patient we identified with the c.859C > G variant was asymptomatic.

In conclusion, our results indicate that the combination of the two clinical evaluation tools increases the chance to detect neonatal neurological signs and to define different early patterns of involvement. This aspect is very relevant since the CHOP INTEND and the HINE2 scales are not age-appropriate neonatal tools, as both had been validated in infants and toddlers [13, 20] including a number of items (head and trunk control reaching, etc.) that are not appropriate for newborns. Furthermore, neither of them was developed as a neurological assessments as the HINE2 records developmental milestones and the CHOP INTEND is a functional scale therefore using a different construct. Combining the HNNE and the floppy infant module, we were able to find some suboptimal performances in approximately half patients identified through the NBS, including minimal signs. These results are similar to previous findings in presymptomatic patients identified through NBS reporting some clinical symptoms in 5 of the 10 patients included [8]. These results however cannot be easily compared as in the previous paper patients were assessed at the time they started treatment, from day 29 to day 150 [8], and therefore were much older and at higher risk of having developed symptoms than in our cohort.

While overt neurological signs at birth or in the first weeks had already been largely reported as part of the type 0 and type 1.1 phenotypes [21], so far, minimal early signs of SMA have not been systematically assessed, likely due to the lack of NBS extensive programs. Suspect patients generally arrived to clinical observation only at the time severe clinical signs had become obvious. Also, we wonder whether some of the presymptomatic patients included in interventional clinical trials [7] may already have had minor signs, as suggested by the variability in CHOP Intend scores and in the therapeutic outcome in the clinical trials in presymptomatic patients suggest that some of these patients may have had minor signs [7].

Despite our experience was limited to a relatively small number of patients, our results suggest that the combined use of the HNNE and the add on module can help to identify patients with different degrees of suboptimal/abnormal findings and to establish whether minor or major early neurological abnormalities may predict the therapeutic outcome. This may help to set up the right expectations and to at least partly explain the differences in the achievement of future milestones or in the timing of when these are achieved. This could be particularly important for infants with 2 *SMN2* copies identified through the screening who have a more variable outcome compared to those with three or more copies. The two assessments will also be of use for the follow up of the infants in the first months to monitor the progression of the diseases also in response to possible treatments.

## Supplementary information

Below is the link to the electronic supplementary material.

Supplementary file1 (JPG 125 KB)

## Data Availability

All data are within manuscript; data are available upon reasonable request to the corresponding author.

## Abbreviations

CHOP INTEND: Children’s Hospital of Philadelphia Infant Test of Neuromuscular Disorders
HINE: Hammersmith Infant Neurological Examination
HNNE: Hammersmith Neonatal Neurological Examination
NBS: Newborn screening
SMA: Spinal muscular atrophy
SMN: Survival motor neuron
