# Supplementary figures and images for: Neurological assessment of newborns with spinal muscular atrophy identified through neonatal screening
Source: Eur J Pediatr. 2022 May 6;181(7):2821–9. doi: 10.1007/s00431-022-04470-3 (PMC9192449; doi:10.1007/s00431-022-04470-3)

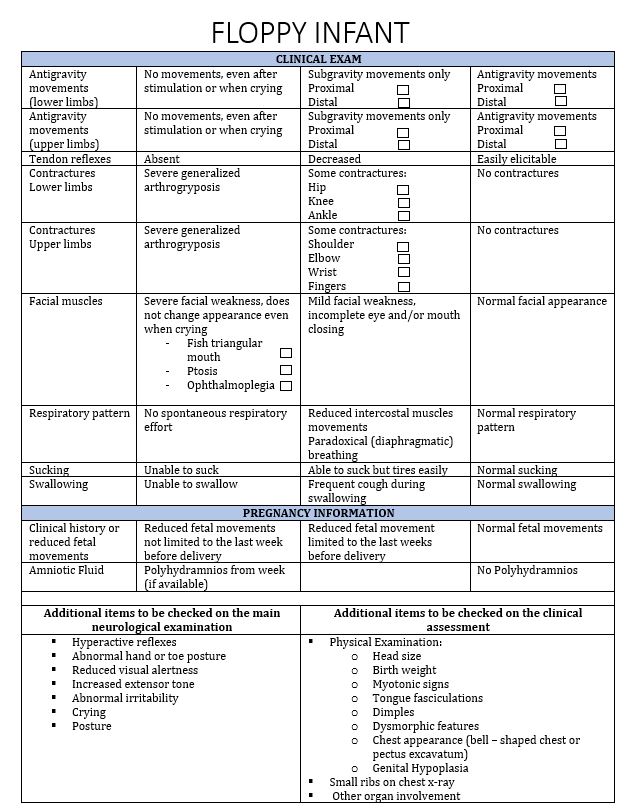

Supplement: Supplementary file 1 — Supplementary file1 (JPG 125 KB) [file 431_2022_4470_MOESM1_ESM.jpg]
